# Autophagy‐associated non‐coding RNAs: Unraveling their impact on Parkinson's disease pathogenesis

**DOI:** 10.1111/cns.14763

**Published:** 2024-05-24

**Authors:** Md Sadique Hussain, Ehssan Moglad, Muhammad Afzal, Shilpa Sharma, Gaurav Gupta, G. V. Sivaprasad, Mahamedha Deorari, Waleed Hassan Almalki, Imran Kazmi, Sami I. Alzarea, Moyad Shahwan, Kumud Pant, Haider Ali, Sachin Kumar Singh, Kamal Dua, Vetriselvan Subramaniyan

**Affiliations:** ^1^ School of Pharmaceutical Sciences Jaipur National University Jaipur Rajasthan India; ^2^ Department of Pharmaceutics, College of Pharmacy Prince Sattam Bin Abdulaziz University Al Kharj Saudi Arabia; ^3^ Department of Pharmaceutical Sciences, Pharmacy Program Batterjee Medical College Jeddah Saudi Arabia; ^4^ Chandigarh Pharmacy College, Chandigarh Group of Colleges Mohali Punjab India; ^5^ Centre of Medical and Bio‐allied Health Sciences Research Ajman University Ajman United Arab Emirates; ^6^ Chitkara College of Pharmacy Chitkara University Rajpura Punjab India; ^7^ Department of Basic Science & Humanities Raghu Engineering College Visakhapatnam India; ^8^ Uttaranchal Institute of Pharmaceutical Sciences Uttaranchal University Dehradun India; ^9^ Department of Pharmacology, College of Pharmacy Umm Al‐Qura University Makkah Saudi Arabia; ^10^ Department of Biochemistry, Faculty of Science King Abdulaziz University Jeddah Saudi Arabia; ^11^ Department of Pharmacology, College of Pharmacy Jouf University Sakaka Al‐Jouf Saudi Arabia; ^12^ Department of Clinical Sciences, College of Pharmacy and Health Sciences Ajman University Ajman United Arab Emirates; ^13^ Graphic Era (Deemed to be University) Dehradun India; ^14^ Graphic Era Hill University Dehradun India; ^15^ Centre for Global Health Research, Saveetha Medical College, Saveetha Institute of Medical and Technical Sciences Saveetha University Chennai India; ^16^ Department of Pharmacology Kyrgyz State Medical College Bishkek Kyrgyzstan; ^17^ School of Pharmaceutical Sciences Lovely Professional University Phagwara Punjab India; ^18^ Faculty of Health, Australian Research Centre in Complementary and Integrative Medicine University of Technology Sydney Ultimo New South Wales Australia; ^19^ Discipline of Pharmacy, Graduate School of Health University of Technology Sydney Ultimo New South Wales Australia; ^20^ Uttaranchal Institute of Pharmaceutical Sciences Uttaranchal University Dehradun India; ^21^ Pharmacology Unit, Jeffrey Cheah School of Medicine and Health Sciences Monash University Malaysia Bandar Sunway Selangor Darul Ehsan Malaysia

**Keywords:** autophagy, ncRNA, neuroinflammation, Parkinson's disease, α‐synuclein

## Abstract

**Background:**

Parkinson's disease (PD) is a degenerative neurological condition marked by the gradual loss of dopaminergic neurons in the substantia nigra pars compacta. The precise etiology of PD remains unclear, but emerging evidence suggests a significant role for disrupted autophagy—a crucial cellular process for maintaining protein and organelle integrity.

**Methods:**

This review focuses on the role of non‐coding RNAs (ncRNAs) in modulating autophagy in PD. We conducted a comprehensive review of recent studies to explore how ncRNAs influence autophagy and contribute to PD pathophysiology. Special attention was given to the examination of ncRNAs' regulatory impacts in various PD models and patient samples.

**Results:**

Findings reveal that ncRNAs are pivotal in regulating key processes associated with PD progression, including autophagy, α‐synuclein aggregation, mitochondrial dysfunction, and neuroinflammation. Dysregulation of specific ncRNAs appears to be closely linked to these pathogenic processes.

**Conclusion:**

ncRNAs hold significant therapeutic potential for addressing autophagy‐related mechanisms in PD. The review highlights innovative therapeutic strategies targeting autophagy‐related ncRNAs and discusses the challenges and prospective directions for developing ncRNA‐based therapies in clinical practice. The insights from this study underline the importance of ncRNAs in the molecular landscape of PD and their potential in novel treatment approaches.

## Graphical abstract

Parkinson's disease (PD) progression is linked to the disruption of autophagy, a cellular process essential for maintaining protein and organelle health. Recent studies reveal that non‐coding RNAs (ncRNAs) are crucial in regulating autophagy, offering new insights into the molecular dynamics of PD. These ncRNAs are implicated in key pathological processes of PD, including autophagy regulation, α‐synuclein aggregation, mitochondrial dysfunction, and neuroinflammation.

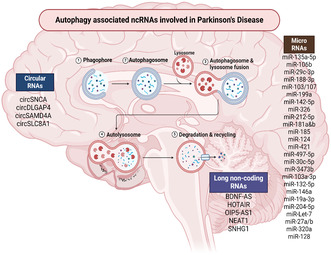

## 1 INTRODUCTION

Parkinson's disease (PD) is a complex neurodegenerative disorder (ND) defined by the gradual deterioration of voluntary movement. This denotes the main clinical characteristic of the illness, and its frequency rises gradually with advancing age.^1^ The manifestations of impaired voluntary motor control include hypokinesia, bradykinesia, akinesia, postural imbalance, stiffness, stooped stance, and shaking at rest. These are often accompanied by gait problems, arm, leg, and trunk rigidity, impaired coordination and sense of balance, and bilateral paralysis of the vocal cords in severe and deteriorating cases. The motor characteristics are utilized to evaluate the reaction to medication and measure the advancement of PD.^2^ PD arises from the demise of dopaminergic neurons (DopN) in the substantia nigra (SN), resulting in a decrease in dopamine levels, usually followed by the buildup of Lewy bodies (LBs) and Lewy neurites in the neurons, with α‐synuclein (α‐syn) complexes being the main protein element.^3^ Co‐occurring pathological alterations, such as excessive tau protein phosphorylation leading to the formation of neurofibrillary tangles, along with the accumulation of amyloid‐β, are often seen in various areas of the brain affected by PD.^4^

Presently, the primary pharmacotherapies for PD encompass dopamine precursors, dopamine agonists (DAs), monoamine oxidase‐B inhibitors (MAOBIs), catechol‐o‐methyl‐transferase inhibitors (COMTIs), anticholinergics, and antiglutamatergics. The diverse mechanisms of action of anti‐PD (APDs) may yield varied effects and clinical utilities. Levodopa‐based formulations, serving as a dopamine adjunct, have demonstrated over 50% enhancement in symptomatic management enduring for 2–3 years.^5^ Nonetheless, prolonged levodopa‐based therapy can precipitate motor complications, including motor fluctuations and dyskinesia.^6^, ^7^ Concurrently, a notable subset of atypical PD patients may gradually develop resistance to levodopa over time.^8^, ^9^ Carbidopa has been associated with various adverse effects including gastrointestinal disturbances and psychiatric manifestations such as heightened alertness, increased motor activity, and elevated aggressiveness, as observed in animal studies. Additionally, there is a risk of weight loss and potential vitamin B6 deficiency, which can lead to complications like seizures, particularly with high doses.^10^, ^11^ Entacapone is linked to gastrointestinal issues such as constipation and related disorders.^12^ Moreover, it can induce drowsiness and pose an elevated risk of seizures, especially in patients with PD who have concurrent vitamin B6 deficiency.^13^ Pramipexole and Rasagiline carry a risk of serotonin syndrome due to potential interactions between antidepressants and anti‐PD with serotoninergic activity, emphasizing the importance of monitoring for this adverse effect in clinical practice.^14^ Currently, available therapies provide relief for symptoms in some groups of patients, but they do not halt the advancement of the illness or undo underlying impairments. Understanding the cause and evolution of diseases is essential for the advancement of diagnostic methods and therapies for PD.

Alongside the excessive accumulation of proteins, there are also malfunctions in breakdown processes, such as autophagy and lysosome processes, which are early signs of the illness and may have a function in the development of PD. This is substantiated by empirical evidence derived from studies on pathology and genomics.^15^, ^16^ The autophagy system is crucial for the prompt elimination of durable proteins and malfunctioning organelles in eukaryotic cells to avert eventual harm and cellular demise. Growing research indicates that the accumulation of α‐syn and tau is an outcome of faulty deterioration via the autophagy‐lysosomal pathway (ALP).^17^ Furthermore, research has shown that α‐syn and tau also affect the activities of mitochondria, autophagy, and lysosomes.^17^, ^18^ DopNs possess a heightened rate of metabolism and need a substantial amount of energy derived from mitochondria. As a result, they are very vulnerable to issues with removing damaged mitochondria.^19^ The buildup of faulty mitochondria leads to higher quantities of reactive oxygen species (ROS), which may harm nearby functional mitochondria and intensify the course of the illness in a self‐perpetuating loop. Wang et al. have shown that VMP1, a protein involved in autophagy, serves a vital function in preserving the balance of neurons and avoiding the deterioration of axons. This suggests that a lack of VMP1 might potentially lead to neurodegeneration (NDG) via the autophagy process. Moreover, Lv et al. established a connection between the control of autophagy and the inflammatory development of PD. The findings of this investigation indicated that microRNA‐3473b hinders autophagy and participates in the regulation of PD.^20^ Furthermore, research suggested that autophagy serves as a shared target of Parkinsonian dysfunctions and emphasized its involvement in the merging of etiology and genetics in the development of PD.^21^

**FIGURE 1:**
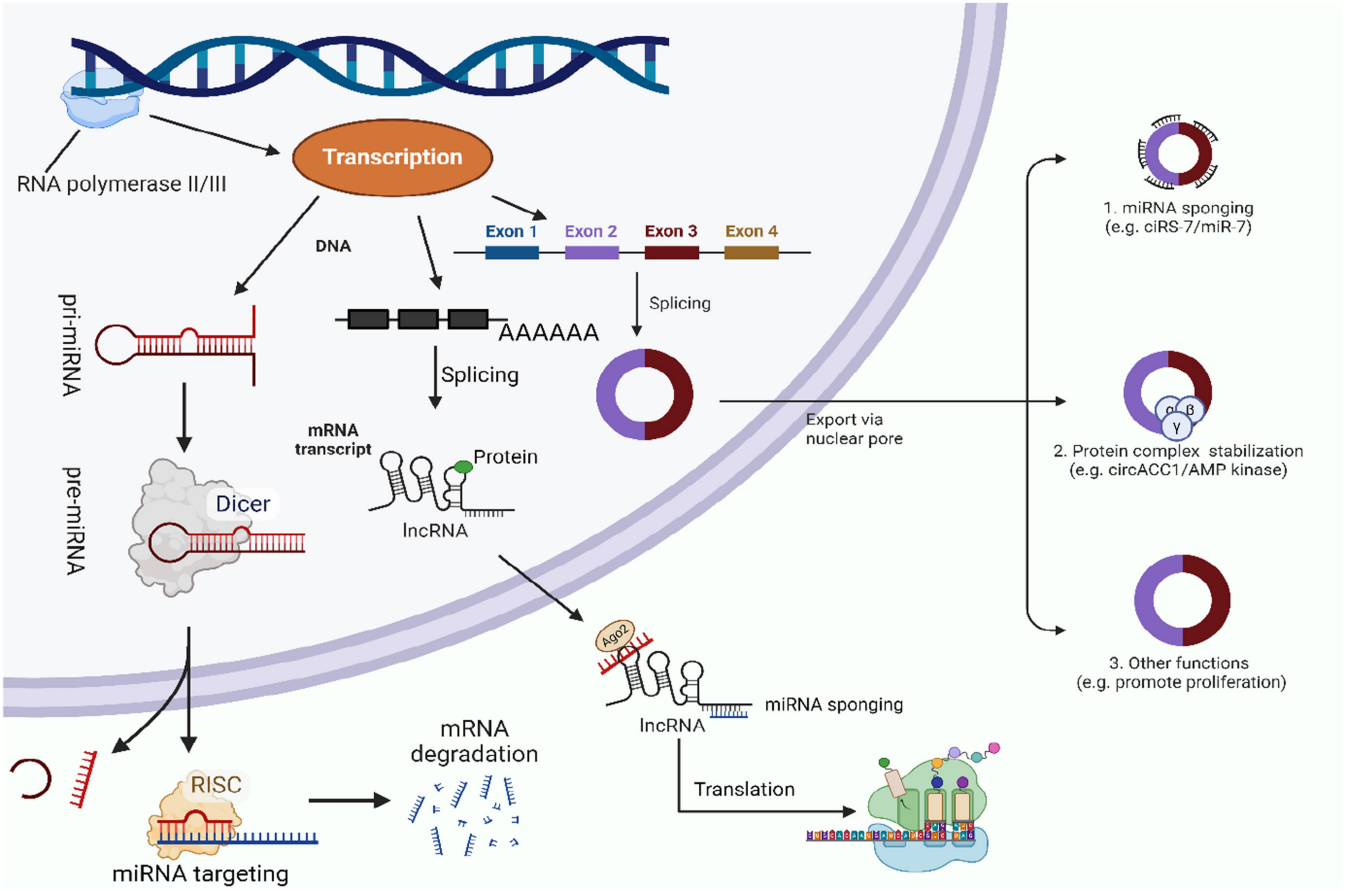
Overview of ncRNAs biogenesis and functions.

Non‐coding RNAs (ncRNAs), including microRNAs (miRNAs), long non‐coding RNAs (lncRNAs), and circular RNAs (circRNAs), exhibited to have important functions in controlling autophagy in many illnesses.^22^, ^23^ Figure 1 illustrates the biogenesis and functions of ncRNAs. Recent research has shown that autophagy may participate in the growth and spread of different tumors. New research findings have shown that ncRNAs play a vital part in controlling the transcription of mRNA and other networks participate in autophagy. These ncRNAs offer tremendous potential in facilitating the progression of illnesses and influencing treatment response.^24^, ^25^ For instance, research has shown that miRNAs and lncRNAs can impact the functioning of autophagy‐related genes (ARGs) and participate in the modulation of autophagy in various conditions.^25^ The contribution of ncRNAs in regulating autophagy is intricate and is anticipated to shift based on the individual cell and illness type.^26^ Comprehending the operational processes of ncRNAs in the control of autophagy may provide novel treatment approaches and targets for a range of illnesses.^24^, ^27^

Investigating ncRNAs related to autophagy in PD is essential for understanding the intricate molecular processes involved in this ND. The disruption of autophagy has been linked to the initiation of PD, and ncRNAs have been identified as important controllers of autophagic mechanisms. Exploring the impact of particular ncRNAs on autophagy in PD has great potential for discovering novel avenues for therapy and indicators for diagnosis. Examining the correlation between autophagy‐associated ncRNAs (AAncRNAs) and processes connected to PD might offer insightful details on the progression of the disease and help identify possible strategies to reduce NDG. This discovery has an opportunity to enhance our awareness of the causes of PD and facilitate the creation of specific treatments that focus on repairing the balance of autophagy in the afflicted areas of the brain. The overview intends to thoroughly examine and combine the existing knowledge on the functions of ncRNAs in regulating autophagy and their influence on the development of PD. The article seeks to assess current research results on the effect of AAncRNAs on the course of autophagy and their contribution to the emergence and advancement of PD.

## 2 AUTOPHAGY IN PARKINSON'S DISEASE

Neurons, being postmitotic in origin, are especially prone to the buildup of faulty organelles and proteins. Therefore, basal autophagy is crucial for preserving neuronal balance. The significance of autophagy for the central nervous system (CNS) was validated through investigations that demonstrated the occurrence of similar characteristics to NDs, such as progressive motor deficits and intracytoplasmic inclusion bodies when autophagy was suppressed in mice lacking the ATG7 and ATG5 genes.^28^ This finding corroborates previous research indicating the significance of autophagy in eliminating proteins that tend to form aggregates in investigations of PD.^29^ Corroborating this, both in vivo and in vitro investigations have reported that autophagic failure is very probable in the development of the illness.

The disruption of autophagy contributes to the development of PD through diverse mechanisms. Dysfunction in these pathways results in the buildup of protein aggregates, a characteristic feature of PD.^30^ Iron dysregulation, particularly the accumulation of iron in the brain, is associated with PD and can interact with α‐syn, a protein present in LBs that is primarily degraded through the autophagy‐lysosome pathway. Ferritin, an iron‐containing protein, is proposed to link iron accumulation to autophagy impairment in PD.^31^ Variants in the GBA gene, which encodes the lysosomal enzyme glucocerebrosidase (GBA), have been associated with a malfunctioning autophagy‐lysosome system and a subsequent reduction in autophagy‐mediated turnover of α‐syn, representing significant risk factors in PD development. In GBA1‐related PD, impaired lysosomal clearance of autophagic substrates and aggregate‐prone proteins is linked to decreased GBA activity and the buildup of α‐syn species.^32^ S‐nitrosylation of the autophagic adaptor protein p62 inhibits autophagic flux, leading to the intracellular accumulation of misfolded proteins. This accumulation may contribute to the spread of aggregated α‐syn in PD and LB dementia.^33^

The forkhead‐box O (FOXO) transcription variables fulfill a vital function in governing the transcription of autophagy.^34^ FOXO3 interacts with and controls a group of ARGs in adult brain stem cells.^35^ Additionally, it induces FOXO1‐mediated autophagy via the activation of the AKT1 cascade.^36^ Dimitriu et al. (2012) reported that FOXO1 was markedly elevated in the prefrontal cortex of people with PD utilizing a transcriptome‐scale microarray method. Furthermore, a significant fraction of genes that possess FOXO1‐attaching regions were also shown to be increased in the corresponding brain region.^37^ The results of this work align with a previous microarray investigation conducted by Zhang et al.,^38^ where FOXO1 consistently exhibited increased expression. The expression statistics for FOXO3 are paradoxical. The existence of LBs throughout the cerebral cortex of PD patients has been strongly linked to increased function and expression of FOXO3a.^39^ Conversely, there has been a notable decrease in the expression of FOXO3 in the brain of persons with PD.^40^ Notwithstanding this lack of uniformity, the involvement of FOXO3 in NDG has been empirically validated by functional investigations conducted on transgenic cell models that produce several forms of the gene, including wild‐type, constitutively active, and dominant‐negative variations. Additional endeavors are required to get an enhanced awareness of the role of FOXO3 in the advancement of PD.

IRE1, the inositol‐requiring enzyme 1α/β, is another possible component that may be involved in regulating autophagy in PD. IRE1 establishes a direct connection between the buildup of proteins and the deterioration of cells. Existing research indicates that IRE1 triggers neuronal cell death (CD) in a PD mouse model via a process that relies on autophagy. In contrast, inhibiting the IRE1 and ATG7 genes halts the advancement of α‐Syn‐mediated PD in the identical laboratory paradigm.^41^

Mitophagy is a particular sort of autophagy that directly breaks down mitochondria. Cell degeneration and CD occur as a result of defective mitochondrial cleaning. The PINK1/Parkin stimulation channel is the most well‐investigated process responsible for mitochondrial breakdown; however, it is not the only possible cause of mitophagy.^42^ When defective mitochondria are detected, PINK1 gathers on the external surface of the mitochondria's membrane and triggers the activation of Parkin (PRKN). It subsequently stimulates the process of ubiquitination in mitochondria, which designates them for removal. Heightened expression of PINK1 confers anti‐apoptotic properties in cells under stress, but its insufficiency renders cells susceptible to stress‐mediated CD. PINK1 has several functions in mitochondria, including the control of mitochondrial membrane potential, the functioning of complex I and IV, as well as ATP and ROS generation. Notably, PRKN stands out for its capacity to provide cellular defense against a diverse range of harmful substances, and its gene expression is increased in response to several forms of stress.^43^ Recessive types of familial PD are often linked to abnormalities in the PINK1 and PRKN genes.^44^ Impairment of either of them results in a reduction in the buildup of mitochondria. Furthermore, the post‐translational changes of PRKN serve an important part in shaping its ability to dissolve or accumulate, as well as its possible involvement in the creation of LBs. Autophagy dysregulation significantly contributes to PD pathogenesis through various mechanisms, including iron interaction, defects in the autophagy‐lysosome system, and protein misfolding accumulation. Strategies targeting autophagy‐lysosome pathways and the development of therapies to enhance lysosomal biogenesis and autophagy may offer promising avenues for discovering disease‐modifying treatments for neurodegenerative disorders.^45^

### 2.1 Significance of autophagy dysfunction and α‐synuclein in PD

The primary changes seen in PD are the buildup of LBs and the degeneration of DopNs in the SN, resulting in a decrease in dopamine synthesis within the brain. LBs, which consist of α‐Syn agglomerates, are utilized as an indicator for α‐synucleinopathies that are characteristic of PD. These LBs are often detected in the SN, and as the illness progresses, they become more widespread across the brain.^46^ There are two primary ideas about the degeneration of DopNs in the SN. The early factor is linked to the existence of α‐Syn clumps, which are often detected in individuals with PD. The second factor indicates harm caused by malfunctioning mitochondria.^47^

The α‐Syn development inside DopNs has been proposed as a process associated with the advancement of the illness since it induces neurotoxicity in the recipient cells. The secretion of this substance occurs in response to stress, lysosomal breakdown, accumulation, proteasome restriction, and mitochondrial malfunction. Being released from the cell is also contingent upon its unique conformation.^48^ The number of damaged neurons will be determined by the generation, accumulation, absorption, and breakdown speed of α‐Syn within and outside cells.^49^ The protein is delivered by processes such as endocytosis, plasma membrane piercing, or exosomes.^48^ Multiple pathways for the absorption of α‐Syn have been proposed.^50^ Previous studies have proposed that LAG3 exhibits a strong attraction to α‐Syn.^49^ However, separate research observed no presence of LAG3, and the excessive or insufficient levels of LAG3 did not impact the α‐Syn disease in A53T mice.^51^ It is worth mentioning that toll‐like receptors (TLRs), namely TLR2 and TLR4, are not functioning properly in individuals with PD. There was a suggestion of a link between the expression of these TLRs and the clustering of α‐Syn.^52^ Furthermore, prior research has shown that the buildup of α‐Syn might initiate the stimulation of NLRP3‐type inflammation.^53^ The involvement of NLRP3 in regulating neuroinflammation and autophagy in PD has been shown.^54^

Multiple studies suggest that α‐Syn has the potential to impact autophagy. According to the findings, the excessive production of α‐Syn enhances the connection between Bcl2 and BECN1, hence suppressing autophagy.^55^ Furthermore, α‐Syn suppresses microglia autophagy.^56^ Studies have shown that α‐Syn causes the buildup of PRKN and interferes with the process of autophagy by inhibiting Rab1, which leads to the incorrect positioning of ATG9.^57^ Furthermore, it has been discovered that the existence of α‐Syn hinders the process of autophagolysosome development and reduces the quantity of SNAP29, responsible for facilitating the fusion of autophagolysosomes.^58^, ^59^ Furthermore, the excessive production of α‐Syn protein resulted in the disturbance of both the structure and arrangement of lysosomes.^60^ It has been discovered that the process of autophagy is hindered and the buildup of misfolded α‐Syn is encouraged by the nitrosylation of the autophagic receptor protein SQSTM1/p62. This alteration enhances the release and dissemination of clustered syn, hence contributing to the suppression of autophagy, injury to neurons, and the distribution of α‐Syn.^61^

The destruction of α‐Syn clusters relies on autophagy. Overall, monomers are broken down by chaperone‐mediated autophagy (CMA),^62^ whereas autophagy participates in the breakdown of aggregates.^63^ However, the specific degradation process also relies on the posttranslational alteration of the proteins.^18^, ^62^ However, various alterations such as α‐Synuclein modifications, phosphorylation, ubiquitination, nitration, oxidation, and posttranslational alterations triggered by dopamine have been seen in cytosolic components in the cerebral cortex of persons with PD and animal models. These alterations have been found to hinder the process of autophagy.^64^, ^65^ Numerous variations in ATG genes may result in diverse symptoms of PD.^66^ Furthermore, it has been shown that elevated concentrations of α‐Syn inside cellular structures, caused by the repression of the GTPase Rab1A, hinder the growth of omegasomes by impacting the location of ATG9.^67^

Given the propagational features of hyperphosphorylated α‐Syn, it has been proposed that its amount diminishes during autophagy. Klucken et al. have provided data supporting the role of the ALP in the breakdown of α‐Syn. The harmful influence of accumulated α‐Syn was increased by the blocking of ALP using bafilomycin A1 (BafA1). However, a decrease in toxicity was seen when α‐Syn clumping was decreased, indicating that protein aggregation could be a process of detoxification.^68^ Recent research has shown that inhibiting ALP with BafA1 led to a decline in the buildup of α‐Syn inside cells and a spike in the release of smaller oligomers. This phenomenon exacerbated biological reactions, including absorption, inflammation, and harm to the cells. Exosomes and RAB11A‐associated processes were responsible for the emergence of α‐Syn in a state of low aggregation, while membrane shedding was responsible for the release of high‐aggregated α‐Syn. This research proposes that the dysfunction of ALP hinders the breakdown of misfolded proteins inside cells, resulting in a greater discharge and spread of α‐Syn in the brain.^69^ Several articles have also acknowledged the significance of autophagy in the capacity of glial cells to eliminate neurotoxic α‐Syn.^70^ Astrocytes have been shown to internalize extracellular α‐Syn produced by neurons and contribute to its propagation.^71^, ^72^ Previous research has proposed that autophagy serves a crucial part in the ability of astrocytes to eliminate and break down harmful substances.^73^ In addition, Tsunemi et al. found that astrocytes had a greater ability to break down α‐Syn than neurons. Moreover, when astrocytes and neurons are cultured together, the transmission of α‐Syn between neurons is reduced.^72^

## 3 NCRNAS AS REGULATORS OF AUTOPHAGY IN PD

NcRNAs refer to a group of RNA transcripts that are unable to produce proteins. Recently, the advancement of genome and structural investigation has led to an acceleration in our knowledge of ncRNAs, thanks to tools like next‐generation sequencing. RNAs can be derived from the majority of genomes. Nevertheless, a mere 2% of it may finally be converted into proteins.^74^ NcRNAs are subdivided into structural and regulatory ncRNAs. Structural ncRNAs are the significant genes responsible for maintaining the fundamental operations of nearly all organisms.^75^ Simultaneously, the preservation of regulatory ncRNAs is less effective compared to structural ncRNAs, which include tiny ncRNAs like miRNAs and lncRNAs. This discovery aligns with the inference that ncRNAs account for the intricacy of multicellular creatures. Traditionally referred to as the “dark transcript” or “genomic dark matter,” ncRNA has been shown to have an essential function in modulating biological processes and activities at both the genetic and epigenetic stages.^76^, ^77^ For example, ncRNAs can guide DNA creation and reorganize the genetic material, as well as shield genes from external nucleic acids.^78^ The role and mechanisms of action of ncRNAs have garnered significant attention lately. While the existence of certain fundamental processes, such as competitive endogenous RNA (ceRNA), has been shown, our comprehension of several alternative pathways remains incomplete.

The brain harbors many forms of ncRNAs including circRNA, lncRNA, and miRNA that have crucial functions in the development, differentiation, arrangement, functioning, and safeguarding of the CNS during the whole lifespan. Through rigorous experimental investigations into the fundamental processes, research findings provide compelling evidence that ncRNAs can control multiple signaling networks linked to NDG. These channels encompass apoptosis, malfunctioning mitochondria, modified protein processing, neuroinflammation, oxidative stress (OS), and the formation of specific protein aggregates.^79^, ^80^ Certain lncRNA, miRNA, and circRNA molecules may also have a role in regulating target proteins and impacting NDG. They do so by participating in the ceRNA process, which operates at both the transcriptional and post‐transcriptional stages. Additional proteins may be regulated directly by others, while neurons can exert control over some proteins by the secretion of exosomes. Despite extensive studies on the molecular cause of PD, the key aspects of its development and advancement are still not precisely understood.^81^

### 3.1 miRNAs

Previous research has shown that miRNAs have a crucial function in controlling ARGs and signaling networks. Consequently, any inaccurate regulation of miRNAs may impact the progression of PD by influencing autophagy. This relationship is shown in Figure 1 and has been supported by findings.^82^, ^83^

#### 3.1.1 MiRNAs' role in neuroprotection through autophagy modulation

miRNAs are being shown to be involved in the pathogenic mechanism of PD by triggering autophagy.^82^, ^83^, ^84^ For example, the level of miR‐326 reduces when PINK1, a gene associated with PD, is not present.^82^ Injecting a miR‐326 replica into mice injected with 1‐methyl‐4‐phenyl‐1,2,3,6‐tetrahydropyridine (MPTP) reduces the amounts of α‐syn and stimulated nitric oxide (NO) synthase and alleviates the movement difficulties of the animals by enhancing the breakdown of DopNs via triggering of JNK network by blocking XBP1.^83^, ^85^ The removal of clumped α‐syn is likewise facilitated by miR‐4813‐3p. miR‐4813‐3p is reduced in a genetically modified Caenorhabditis worm model of PD, wherein a high level of α‐syn leads to OS in neurons. miR‐4813‐3p activates protein quality monitoring mechanisms, such as the ALP and the ubiquitin‐proteasomal network, to remove misfolded and clumped proteins. This indicates that addressing miR‐4813‐3p might be beneficial in treating PD.^86^ In addition, the transport of miR‐106b via extracellular vesicles derived from mesenchymal stem cells (MSCs) helps to prevent CD by MPTP. It also improves the process of autophagy by declining the activity of CDKN2B, a gene responsible for producing a protein that causes cell cycle stoppage in the G1 stage. This is followed by an upsurge in BCL2 and a decline in BAX.^87^, ^88^ Sun et al.^89^ showed that miR‐212‐5p appears at minimal levels in SH‐SY5Y cells and the midbrain of PD mice. They found that injecting miR‐212‐5p imitates into the midbrain reduces the degradation of DopNs. This effect is achieved by blocking sirtuin2 and triggering autophagy, which is accomplished by lowering cytoplasmic p53 expression. Furthermore, the N‐methyl‐4‐phenylpyridinium (MPP+)‐infected SH‐SY5Y cells and MPTP‐infected mice exhibited a reduction in the expression of miR‐124, along with an increase in autophagosomes and a decline in lysosomes. Increasing the expression of miR‐124 by the use of substances that activate it and imitate its function reduces the degeneration of DopNs and increases the amount of dopamine in the striatum by repairing the disrupted autophagy. This is accomplished by concurrently suppressing the synthesis of BIM and preventing the movement of BAX to the mitochondria.^90^, ^91^ Additional examination of the defensive strategy of miR‐124 has shown that introducing miR‐124 into the SN of mice exposed to MPTP hampers the expulsion of inflammatory mediators from stimulated microglia by repressing the expression of p62 and p38.^92^ The results suggest that reduced activity of miRNAs in the brains of PD mice is linked to the buildup of α‐syn, OS, neuronal CD, and neuroinflammation. Conversely, increasing the levels of these miRNAs mitigates these consequences by promoting autophagy. Hence, the regulation of autophagy using miRNAs has great potential as a novel treatment approach for PD.

According to reports, miR‐142‐5p is decreased in SH‐SY5Y cells when they are exposed to 6‐OHDA. Increasing the level of miR‐142‐5p improves the health of neurons by inhibiting autophagy that depends on Beclin‐1 (bcl1). Based on this, miR‐142‐5p serves a defensive function in the advancement of PD.^93^ Furthermore, miRNAs have been involved in the control of the autophagy‐related signaling system (ARSS) in PD, despite addressing ATGs. For instance, the amount of miR‐199a is reduced in PC12 cells administered with MPP+. However, introducing miR‐199a replicas by transfection enhances the longevity and survival of neurons by promoting the PTEN/AKT/mTOR cascade.^94^, ^95^ In a similar setting, overexpressing miR‐181b activates this signaling system, leading to the subsequent suppression of autophagy. This suppression helps to reduce the cytotoxic effects of MPP+.^96^ MiR‐135a‐5p inhibits the mTOR/ULK1/S6K1 cascade in MPP+‐injected SH‐SY5Y and CHP‐212 cells, reducing MPP+‐mediated neuronal CD.^97^ Similarly, the level of miR‐185 is reduced in SH‐SY5Y cells grown with MPP+. Conversely, increasing the expression of miR‐185 suppresses CD via deactivating the AMPK/mTOR network.^98^, ^99^ Similarly, MPP+ reduces the level of miR‐181a in SK‐N‐SH cells. Conversely, increasing the level of miR‐181a lowers the pace of CD by preventing the induction of the p38MAPK/JNK system.^100^ The findings indicate that miRNAs have a defensive function in the advancement of PD by deactivating autophagy by addressing several ARSS. Furthermore, it should be emphasized that miRNAs can influence the conclusive phase of autophagy by controlling the cyclin‐dependent kinase 5 (CDK5) system, shown to cause CD triggered by MPTP.^101^ Li et al.^102^ showed that autophagy is inhibited in both MPP+‐exposed MN9D cells and MPTP‐exposed mice by reducing the levels of miR‐103/107 and stimulating the CDKR5 cascade. Additionally, HMGA1 is increased to maintain the expression of miR‐103/107, creating an adverse feedback cycle between HMGA1 and miR‐103/107. This cycle contributes to neural protection by managing autophagy. Furthermore, the administration of exosomes loaded with miR‐188‐3p, obtained from adipose‐derived cells, into mice treated with MPTP, effectively inhibits CDK5‐mediated autophagy. This inhibition leads to the prevention of α‐syn accumulation, NLRP3‐driven inflammasomes, and neuronal injury in the SN.^103^ These data suggest that targeting CDK5‐induced autophagy may represent a promising approach to the management of PD. Furthermore, investigations have demonstrated that miR‐29c‐3p may suppress the induction of microglial NLRP3 inflammasome and the incidence of apoptosis in neurons in models of PD.^104^ Additional mechanistic analysis has shown that the increase in miR‐29c‐3p suppresses autophagy by reducing the levels of 10–11 translocation 2. As a result, this alleviates the degeneration of DopNs in the SN caused by MPTP.^105^, ^106^

Neuroprotective miRNAs are reduced in different animal and cellular forms of PD. Increasing the amount of these miRNAs may mitigate α‐syn pathology, neuroinflammation, neuronal OS, and CD. This is followed by changes in autophagy stages, either increasing or decreasing it, by focusing on ATGs and ARSS (Table 1). As previously stated, it is well recognized that increased autophagy may effectively slow down the course of PD in the majority of instances due to its neuroprotective characteristic. Nevertheless, several neuroprotective miRNAs are linked to reduced degrees of autophagy. It is worth mentioning that a similar miRNA seems to have neuroprotective impacts on the course of PD, depending on the amount of autophagy. Using miR‐124, multiple investigations have shown that it induces autophagy to prevent microglial triggering and neuronal demise in both living organisms and laboratory settings.^90^, ^92^ However, another investigation has shown that miR‐124 inhibits CD.^107^, ^120^ The precise mechanics of these phenomena are yet unknown. PD stimuli, like MPP+, have been seen to activate autophagy in a way that depends on their intensity. Excessive autophagy, either by high concentrations or extended induction by PD toxins, may lead to autophagic CD.^94^ Therefore, the increased expression of these miRNAs may serve as a protective response to prevent autophagic neuronal CD. Prospective studies should examine the amount of PD‐associated stimulus and the various PD models. This is because the dosage of PD stimulus therapy may impact the control of miRNAs in the context of autophagy throughout the progression of illness. Therefore, subsequent studies should assess the difference in concentration of the PD stimulus to determine the most effective concentration for simulating PD pathophysiology. Furthermore, the administration of these neuroprotective miRNAs to individuals with PD could offer a promising treatment approach via regulating autophagy. Currently, the strategy of using locked nucleic acids altered oligonucleotides to address miR‐122 is being tested in stage I clinical trials for hepatitis C.^121^, ^122^ Additional investigation into therapeutic strategies using miRNAs, such as utilizing nanoparticles (NPs) encapsulated with miRNA and microinjection of miRNA, is anticipated to be effective in treating PD.

**TABLE 1:** The neuroprotective and neurotoxic influence of microRNAs that regulate autophagy in Parkinson's disease.

miRNAs	Expression	Role	Autophagy	References
miR‐135a‐5p	↓	Neuroprotective	−	97
miR‐106b	↓	Neuroprotective	+	87
miR‐29c‐3p	↓	Neuroprotective	−	105
miR‐188‐3p	↓	Neuroprotective	−	103
miR‐103/107	↓	Neuroprotective	−	102
miR‐199a	↓	Neuroprotective	−	94
miR‐142‐5p	↓	Neuroprotective	−	93
miR‐326	↓	Neuroprotective	+	83
miR‐212‐5p	↓	Neuroprotective	+	89
miR‐181b	↓	Neuroprotective	−	96
miR‐185	↓	Neuroprotective	−	98
miR‐181a	↓	Neuroprotective	−	100
miR‐124	↓	Neuroprotective	+	92
miR‐124	↓	Neuroprotective	+	90
miR‐124	↓	Neuroprotective	−	107
miR‐421	↑	Neurotoxic	−	108
miR‐497‐5p	↑	Neurotoxic	−	109
miR‐30c‐5p	↑	Neurotoxic	−	110
miR‐3473b	↑	Neurotoxic	−	20
miR‐103a‐3p	↑	Neurotoxic	−	111
miR‐132‐5p	↑	Neurotoxic	+	112
miR‐146a	↑	Neurotoxic	−	113
miR‐19a‐3p	↑	Neurotoxic	−	114
miR‐204‐5p	↑	Neurotoxic	−	115
miR‐Let‐7	↑	Neurotoxic	−	116
miR‐27a/b	↑	Neurotoxic	−	117
miR‐320a	↑	Neurotoxic	−	118
miR‐128	↓	Neurotoxic	−	119

*Note*: ↓, Downregulation; ↑, Upregulation; +, Activation, −, Inhibition.

#### 3.1.2 Neurotoxic impacts of miRNAs via the modulation of autophagy

While miRNAs have demonstrated neuroprotective effects in PD, enhancing their levels has been associated with disease worsening via inhibiting autophagy. Research has indicated that miR‐204‐5p is abundantly present in both the bloodstream and neural cells of PD mice triggered by MPTP. This molecule increases the concentrations of α‐syn and tau in the SN, leading to a disruption of the autophagy managed by ATG7 and the apoptotic network initiated by JNK in DopNs. Consequently, this disturbance results in impaired CD.^115^ The miR‐30c‐5p/ATG5 pathway, which hampers the course of PD, leads to a reduction in antioxidants, dopamine, its byproducts, and CD. This exacerbates motor impairments in mice administered MPTP.^123^ Similarly, the inhibition of miR‐let‐7, which is boosted in a C. elegans model of PD, reduces the production of α‐syn, OS produced by ROS, and motor performance via reducing autophagy linked to ATG13.^116^ Research also indicates that the suppression of miR‐497‐5p, which is highly expressed in models of PD, has a protective effect on SH‐SY5Y cells exposed to MPP+ by preventing CD and enhancing the process of autophagy. This is achieved through the increased expression of fibroblast growth factor‐2 (FGF2).^109^, ^124^, ^125^ These findings suggest that elevated miRNA levels in PD worsen the illness by suppressing neuronal autophagy by attacking different ATGs. However, miRNAs can influence microglial autophagy and hence participate in the inflammatory process in PD. Lv et al.^20^ showed that miR‐3473b is upregulated in the cerebral cells of mice exposed to MPTP, leading to an augmentation of the inflammatory response. Transfecting microglial cells with an miR‐3473b mimic enhances the release of inflammatory cytokines TNF‐α and IL‐1β by reducing the expression of TREM2/ULK1, which in turn hinders the process of autophagy.^20^ Moreover, it is being established that the elevated presence of miR‐19a‐3p in the exosomes of SH‐SY5Y cells that have been genetically modified with the α‐syn gene actively suppresses the process of microglial autophagy. This suppression occurs by specifically addressing the PTEN/AKT/mTOR cascade. As a result, the ability of microglia to engulf and eliminate α‐syn, as well as the development of inflammation, may be negatively impacted. The information presented indicates that miR‐19a‐3p may serve in the neurotoxic effects associated with autophagy in PD.^114^, ^126^

Moreover, the unusual activity of miRNAs is linked to the worsening of PD via the regulation of signaling systems connected to mitophagy. During prolonged mitochondrial stress, miR‐27a/b is increased as a protective response to inhibit the activation of mitophagy by reducing the activity of PINK1, which impairs the breakdown of injured mitochondria by lysosomes.^117^ Mitochondrial autophagy, namely the process of PINK1‐mediated mitophagy, is hindered by miR‐421 in both cells affected by PD and mice. Consequently, the normal functioning of mitochondrial autophagy is disturbed, resulting in the accumulation of ROS and subsequent OS in neurons. Furthermore, in SH‐SY5Y cells subjected to rotenone, miR‐146a is elevated via NF‐kB‐mediated transcriptional stimulation. This rise in miR‐146a leads to a reduction in mitophagy by suppressing PRKN, which in turn causes a buildup of malfunctioning mitochondria and excessive formation of ROS in deteriorating neurons.^113^ Consistently, reducing the levels of miR‐103a‐3p, an miRNA that is abundantly present in models of PD generated by MPTP, reduces the degradation of DopNs in the SN and enhances walking difficulties by initiating the process of mitophagy mediated by the PRKN/Ambra1 pathway.^111^, ^127^ Therefore, our findings demonstrate that the impairment of mitophagy caused by miRNA is a crucial pathological mechanism in PD. In addition, it has been shown that increased concentrations of miR‐320a in PD brains inhibit CMA by addressing heat shock protein 70, which promotes the buildup of α‐syn inside cells.^118^, ^128^

The investigation indicates that the majority of the miRNAs discussed are increased in PD models and contribute to neurological damage by participating in different pathological processes, such as the buildup of α‐syn, malfunction of mitochondria, and triggering of microglia through the inhibition of autophagy (Table 1). On the other hand, conflicting research shows that blocking miR‐132‐5p, which is increased in PD models generated by MPTP, reduces CD and the process of autophagy by directly affecting ULK1. This suggests that autophagy is increased in the neurotoxic effects of miR‐132‐5p in PD.^112^ One possible reason for this might be because miR‐132‐5p plays a role in the excessive stimulation of autophagy in neurons caused by MPTP, which eventually results in CD by autophagy. Furthermore, miRNAs modulate autophagy via intercommunication with apoptotic CD. For instance, prior research has revealed that overexpression of bcl1 by miRNAs increases the effectiveness of autophagy while also inhibiting apoptosis.^30^, ^129^ Therefore, it is crucial to have a deeper knowledge of how miRNA regulates autophagy to fully comprehend the development of PD. In addition, these neurotoxic miRNAs may impact the course of PD via many targets. Using miR‐30c‐5p as a case study, it has been observed that the administration of miR‐30c‐5p antagomir reduces neuronal CD and triggers autophagy in the neural tissue of mice subjected to MPTP.^123^ Additionally, multiple investigations have shown that miR‐30c‐5p can also regulate immune responses and pyroptosis, both of which are linked to PD advancement.^110^, ^130^ Hence, further studies are necessary to definitively identify the primary target of miRNAs in PD. Alternatively, miRNAs have contrasting impacts on PD. For example, the level of miR‐128 is reduced in rats with PD, and increasing its level helps prevent CD, thereby protecting against PD.^131^ Nevertheless, miR‐128 functions as an inhibitory controller of transcription factor EB, a crucial controller of the autophagy‐lysosome system. Consequently, it hinders the elimination of α‐syn oligomers, exacerbating the toxicity of α‐syn in PD.^119^, ^132^ The combination of neurotoxic miRNA regulation and numerous pathogenic events makes the control of autophagy in the course of PD complex. The relationship between miRNA‐controlled autophagy and the development of PD has not been completely understood.

### 3.2 lncRNAs

**FIGURE 2:**
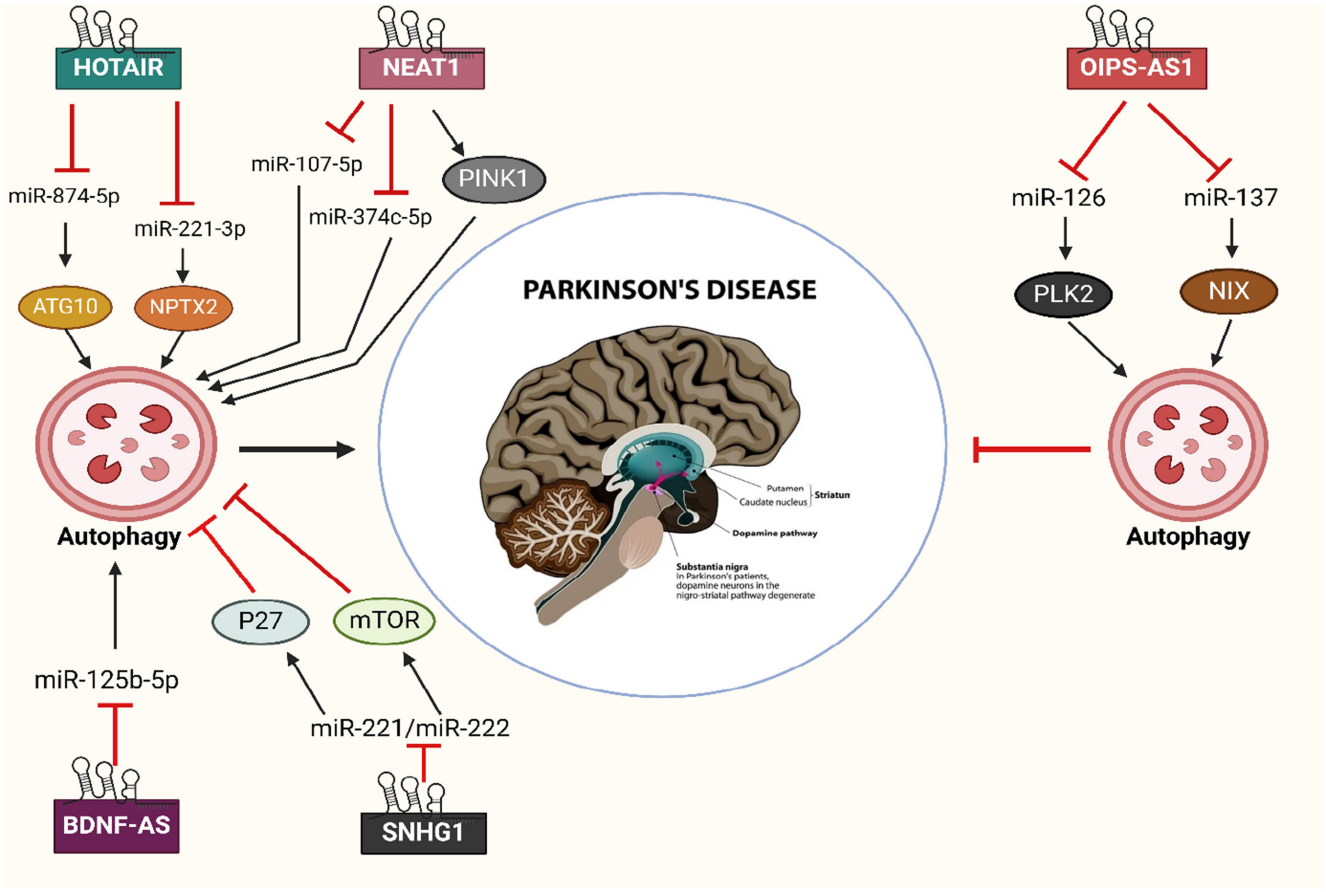
LncRNAs possess an effect in the development of Parkinson's disease via influencing the process of autophagy.

lncRNAs have garnered heightened study attention, particularly concerning NDG in brain activity and CNS illnesses. A multitude of lncRNAs have been shown to have aberrant expression in the cytoplasm in PD (Figure 2 and Table 2). As a first study on the importance of lncRNAs in PD, Kraus conducted a thorough examination of the expression levels of 90 well‐documented lncRNAs in 30 cerebral cortex samples from 20 PD individuals and 10 healthy subjects. It was discovered that the H19 upstream conserved 1 and 2 are considerably reduced in PD. Furthermore, the expression levels of lincRNA‐p21, LINC‐PINT, MALAT1, SNHG1, and tiny ncRNAs are notably increased.^139^ Furthermore, a microarray study has shown that the increased expression of HOTAIRM1 and AC1131056.3, as well as the decreased expression of XIST, is involved in the worsening of PD by causing damage to DopNs.^140^, ^141^ Moreover, the atypical accumulation of α‐syn has been linked to the role of lncRNAs. NEAT1 was discovered to speed up the aggregation of α‐syn and at the same time enhance the process of programmed CD (PCD) in PD mice caused by MPTP.^142^ Lin et al.^79^ discovered that lncRNA G069488, RP11‐142 J21.2, and AC009365.4 are linked with the buildup of α‐syn in cells produced by α‐syn oligomers, as shown by microarray testing.

**TABLE 2:** The involvement of autophagy‐linked lncRNAs in the development of Parkinson's disease.

Illness	LncRNA	Role in Autophagy	Function	References
Parkinson	BDNF‐AS	↑	+	133
	HOTAIR	↑	+	134
	OIP5‐AS1	↑	−	135, 136
	NEAT1	↑	+	137
	SNHG1	↓	+	138

*Note*: ↑, Activate; ↓, Inhibit; +, Promote; −, Suppress.

The disruption of autophagy is strongly associated with the buildup of α‐syn aggregates. L1CAM, an exosome originating from neurons, is being shown to be associated with the ALP, indicating its partial involvement in autophagy. Zou et al.^143^ provided evidence that linc‐PIU3F3 has a stimulatory influence on α‐syn in L1CAM. Furthermore, it has been shown that the α‐syn pathogenetic aggregate, namely lncRNA‐T199678, acts as a sponge for miR‐101‐3p to alleviate damage to DopNs.^144^ In addition, Huang et al. and Quan et al. enrolled individuals with PD and healthy individuals to assess the levels of MEG3, which are reduced in individuals with PD. The researchers discovered that increasing the expression of MEG3 in MPP+ SH‐SY5Y cells prevented apoptosis by enhancing the expression of LRRK2.^145^, ^146^, ^147^

Recent investigations indicate that autophagy is an intriguing avenue for the treatment of PD. The pathogenesis of PD is regulated by lncRNAs through their modulation of the autophagy signaling cascade. Several lncRNAs, including NEAT1, HOTAIR, and BDNF‐AS, participate in the worsening of PD via stimulating autophagy. The expression of NEAT1 was substantially elevated by MPTP in PD models. NEAT1 facilitated MPTP‐mediated autophagy by sustaining the PINK1 protein, and the inhibition of NEAT1 reduced autophagy and mitigated damage to DopNs.^137^ It was revealed that NEAT1 directly affected the expression of miR‐374c‐5p. When NEAT1 was silenced, miR‐374c‐5p was elevated, autophagy and apoptosis were inhibited, and the ratio of tyrosine hydroxylase (TH+) neurons climbed in MPTP‐induced PD animals.^148^, ^149^ Similarly, Li et al. discovered a beneficial association between the transcription of NEAT1 and the quantity of MPP+. They also observed that messing with NEAT1 significantly suppressed autophagy and apoptosis in PD mice by increasing the expression of miR‐107‐5p.^150^ The data suggest that NEAT1 serves an essential function in the pathophysiology of PD. It might be regarded as an appropriate focus for the diagnosis and therapy of PD. Recent research has shown that HOTAIR may exacerbate the course of PD. The expression of HOTAIR was elevated in the compact tissues of the SN in the PD model subjected to MPP+. HOTAIR may interact with miR‐221‐3p to increase the expression of NPTX2, a target of miR‐221‐3p. This interaction enhances the process of autophagy in DopNs, both in laboratory settings (in vitro) and in living organisms (in vivo).^134^ Similarly, Zhao et al. discovered that HOTAIR facilitated MPP+‐induced neuronal damage in SK‐N‐SH cells by acting as a sponge for miR‐874‐5p. MiR‐874‐5p specifically inhibited the expression of ATG10, an essential determinant implicated in autophagosome development.^151^, ^152^ The expression of the BDNF‐AS is abnormal in PD. A current study conducted by Fan et al. revealed that BDNF‐AS was also increased in mice with PD produced by MPTP, as well as in DopNs in a cell model of PD created by MPP+ in SH‐SY5Y cells. Suppression of BDNF‐AS resulted in an enormous spike in TH+ neurons, inhibition of autophagy, and enhanced cell survival via the regulation of miR‐125‐5p expression.^133^

In contrast, lncRNAs also have a role in the development of PD by suppressing the process of autophagy. For instance, elevated levels of SNHG1 were detected in postmortem cerebral cortex specimens from PD models. Suppression of SNHG1 facilitated autophagy and hindered neuronal CD mediated by MPP+.^138^ SNHG1 can interact with the miR‐221/222 group, leading to the activation of p27 and mTOR transcription. This, in turn, activates the mTOR system and suppresses autophagy.^138^

The OIP5‐AS1 has been demonstrated to function as a significant controller of regeneration and exhibits a defensive impact for various NDs.^153^ OIP5‐AS1 was reduced in a cellular model of PD treated with MPP+. However, when OIP5‐AS1 was overexpressed, it led to an upsurge in the production of NIX by acting as a sponge for miR‐137. The rise in NIX expression encouraged mitochondrial autophagy and protected against neuronal cell deterioration.^135^ OIP5‐AS1 can decrease the buildup and harmful effects of α‐syn in MPP+‐exposed SH‐SY5Y cells. It does this by concentrating on miR‐126 and increasing the production of PLK2.^136^

### 3.3 circRNAs

CircRNAs are considered significant macromolecules for comprehending and tackling the onset of NDs such as PD.^154^ Research has documented that the activity of circRNAs in the CNS of individuals with PD may vary (Table 3). There is growing data suggesting that circRNAs may serve a significant part in the advancement of PD.^154^, ^156^ Ravanidis et al. (2021) observed changes in the expression patterns of six circRNAs in peripheral blood mononuclear cells from individuals with PD as opposed to healthy volunteers. These circRNAs are circ_0001566, circ_0006916, circ_0000497, circ_0001187, circ_0004368, and circ_0003848. A notable characteristic of PD is the abnormal expression and accumulation of α‐syn, which is seen in LBs. miR‐7 has been shown to limit the expression of α‐syn by attaching to the 3′ UTR of the α‐syn and impeding the translation process.^158^, ^159^ McMillan et al.^160^ noticed a decline in the concentrations of miR‐7 in the SN of individuals diagnosed with PD. The circRNA ciRS‐7 has 63 binding regions for miR‐7, indicating its role in regulating miR‐7. This circRNA is likely to have a crucial part in the functioning of neurons and is also implicated in the growth of brain tumors and NDs.^161^ Additionally, in an additional investigation conducted on PD, researchers found that the removal of α‐syn and associated clumps was aided by miR‐7 via a process called autophagy in specialized ReNcell VM cells. According to the authors, miR‐7 promotes the production of LC3 puncta and enhances the transformation of LC3‐I to LC3‐II, indicating an upsurge in autophagosome development.^162^ Furthermore, miRNAs that concentrate on α‐syn have been displayed as having a favorable impact in protecting against PD models induced by MPP+ or MPTP. The researchers discovered that the increased expression of α‐syn, facilitated by miR‐7, serves a function in the degradation of the nigrostriatal pathway in both cultured cells and animals in the MPTP‐induced neurotoxic model of PD.^163^ Furthermore, the introduction of miR‐7 into the HeLa cell line led to a more efficient suppression of α‐syn despite the lack of circRNA ciRS‐7. This suggests that ciRS‐7 may have an impact on α‐syn expression in a way that depends on miR‐7, and this relationship is also associated with the development of PD.^157^ A further investigation has shown that circSNCA functions as a miR‐7 sponge, leading to an augmentation in the synthesis of α‐syn mRNA in SH‐SY5Y cells. This, in turn, inhibits autophagy and promotes CD. Sang et al. (2018) observed that the reduction of circSNCA resulted in a higher level of LC3B‐II and BCL2, indicating the inhibition of CD and the enhancement of cellular self‐degradation in PD. They also discovered that the administration of a dopamine D2/D3 receptor agonist drastically lowered the levels of SNCA and circSNCA. They found that the downregulation of circSNCA led to a reduction in the manifestation of genes that promote PCD, including P53, PTEN, CASP3, and BAX.^155^, ^164^

**TABLE 3:** Collection of circRNAs that are linked with the processes of autophagy and Parkinson's.

circRNAs	Diseases	Functions	References
circSNCA	Parkinson	Modulates autophagy and cellular apoptosis via modulating the miR‐7/ α‐syn pathway	155
circDLGAP4		Enhances autophagy, suppresses apoptosis, and displays neurological protection via regulating miR‐134‐5p/CREB cascade	156
circSAMD4A		Linked to autophagy and programmed cell death in dopaminergic neurons via regulating miR‐29c‐3p/AMPK/mTOR cascade	157
circSLC8A1		May participate in oxidative stress by acting as a miRNA sponge	154

An important way by which circRNA contributes to the advancement of diseases is by its association with miRNAs related to such diseases.^165^ The function of circRNAs as miRNA decoys in different disorders has also been examined.^166^ Knocking out circRNA zip‐2 in PD resulted in a decrease in the accumulation of α‐syn by acting as a miR‐60 sponge. This contributed to an improved prognosis for people with PD.^167^ Another study found that circRNA s‐7 increased the expression of crucial genes related to PD and Alzheimer's disease (AD) by inhibiting miR‐7.^168^ The downregulation of circHIPK2 expression substantially decreased the activation of astrocytes via modulating autophagy and endoplasmic reticulum (ER) stress via addressing miR‐124–2HG and SIGMAR.^169^ CircDLGAP4 functions as a molecular sponge in people with PD and engages in competition with miR‐134‐5p to impede its operation. It induces the upregulation of BECN1 and LC3‐II, leading to enhanced autophagy, suppression of apoptosis, and reduction of mitochondrial dysfunction. CircDLGAP4 decreases in mice models of PD, perhaps playing a role in PD growth by impacting survival, apoptosis, destruction of mitochondria, and autophagy.^156^ Recent findings indicate that in PD, circSAMD4A disrupts the AMPK/mTOR pathway via miR‐29c‐3p, leading to the demise and autophagy of DopNs. miR‐124 has been demonstrated to safeguard DopNs in PD by controlling the AMPK/mTOR pathway, which regulates PCD and cellular self‐degradation (autophagy).^157^, ^170^

CircRNAs progressively increase with age in the SN of a healthy brain. However, in individuals with PD, the overall count of circRNAs is decreased owing to the disruption of this association. Hanan et al. (2020) reported that the expression of circSLC8A1, with seven binding locations for miR‐128, was higher in the SN of persons with PD. Additionally, higher levels of circSLC8A1 were seen in cultured cells exposed to Paraquat, which is a model for OS. Hanan et al.^154^ also observed a spike in the expression patterns of miR‐128 targets in persons with PD. The aforementioned study has emphasized the significance of AAcircRNAs as promising candidates for therapeutic intervention in PD. There is a limited amount of research on the function of circRNAs in modulating autophagy in PD, so additional analyses are required.

## 4 THERAPEUTIC IMPLICATIONS: TARGETING AUTOPHAGY‐ASSOCIATED NCRNAS IN PD

Considering the capacity of ncRNAs to control the progression of PD by influencing cellular autophagy, directing attention toward autophagy‐related lncRNAs in neurons presents a potential treatment strategy for managing PD. Presently, there are two prominent approaches using oligonucleotides, namely antisense oligonucleotides (ASOs) and RNA interference (RNAi), that have shown effectiveness in decreasing the expression of overactive ncRNAs in neurons. This suggests that RNA‐based treatments offer significant therapeutic possibilities for PD.^171^, ^172^ Nevertheless, the primary hindrance to the CNS is the blood–brain barrier (BBB); oligonucleotides are incapable of traversing the BBB. Recent research indicates that the use of RNA‐based therapeutics in conjunction with liposomes can improve the ability to penetrate the BBB.^173^, ^174^ Cell‐derived exosomes are also regarded as treatment vesicles for delivering RNAs to the CNS. The secondary framework of lncRNAs presents a potential challenge in establishing them as therapeutics. However, this impediment may potentially be addressed by using chemically altered equivalents.^175^, ^176^ Furthermore, the absence of conservation in ncRNA patterns across individuals and experimental animals may result in additional challenges when developing treatments that apply to both.^177^ Nowadays, only a limited number of ncRNAs have been thoroughly investigated for their role in the development of PD via their influence on the autophagy system. Further empirical inquiries are necessary to comprehend the role and processes of aberrantly expressed ncRNAs identified using high‐throughput sequencing in CNS diseases. Given the advancements in RNA‐based treatment approaches, we suggest that ncRNAs have significant potential as a valuable therapeutic resource for PD.

Current therapeutic approaches for PD face several constraints. While medications like L‐DOPA effectively mitigate motor symptoms, they fail to impede disease progression. There is a pressing need for neuroprotective treatments capable of decelerating or halting neurodegeneration.^178^, ^179^ RNA‐based therapies, including ASOs, small interfering RNAs (siRNAs), and miRNAs, may induce off‐target effects, resulting in unintended outcomes.^180^ An extensively researched approach to nucleic acid‐based therapeutics involves employing ASOs. These short, synthetic, single‐stranded oligonucleotides can regulate protein expression through various mechanisms. They can influence pre‐miRNA splicing or interact with mRNA, thereby facilitating the correction of faulty RNA or the removal of proteins associated with disease.^181^, ^182^ The BBB poses a significant hurdle in delivering RNA‐based therapies to the brain. To surmount this obstacle, researchers are developing NPs and other delivery systems.^183^, ^184^ The exorbitant cost associated with developing and manufacturing RNA‐based therapies can hinder patient access.^180^, ^184^ Long‐term safety data concerning RNA‐based therapies remain scarce, necessitating further investigation to comprehensively assess their safety profile. RNA‐based therapies are inherently more intricate than conventional small‐molecule drugs, rendering their development and regulatory approval more arduous. Resistance to RNA‐based therapies is a concern, as cells may develop resistance over time, thereby diminishing treatment efficacy.^180^, ^185^, ^186^ Despite these challenges, RNA‐based therapies show promise in PD and other NDs. Ongoing research endeavors aim to tackle these hurdles and enhance the safety, effectiveness, and accessibility of such therapies.

To tackle the constraints of existing RNA‐based therapies for PD, scientists are exploring various potential remedies. They are devising sophisticated delivery systems like NPs, liposomes, and alternative carriers to surmount the BBB and transport RNA‐based therapies directly to the brain. Researchers are striving to craft RNA‐based therapies that precisely target specific cells, thereby minimizing the risk of off‐target effects.^184^

Recent studies indicate that RNA therapeutics may require low dosages, potentially reducing adverse effects—an issue often encountered in drug development. However, naturally occurring nucleotide‐based therapeutic oligonucleotides face challenges such as rapid in vivo degradation or renal clearance, rendering them unsuitable for drug development. Therefore, synthetic oligonucleotides are commonly employed in RNA‐based technologies. Despite these advancements, chemical modifications may alter the structure of RNA, potentially triggering cellular responses or off‐target effects. Moreover, errors in synthesizing longer sequences can affect functionality or induce cellular toxicity. Additionally, cost considerations and the provision of synthetic RNA in micromolar scales may impact clinical utility.^187^, ^188^ Enzymatic synthesis and recombinant biosynthesis offer alternative production methods. Enzymatic synthesis, utilizing bacteriophage systems, enables RNA production from DNA sequences via in vitro transcription. However, it presents challenges such as heterogeneity and decreased reliability with increasing transcript length. Recombinant biosynthesis involves host cells expressing target RNA sequences from modified plasmid DNA. Challenges include purification difficulties and susceptibility to RNases. Although enzymatic and recombinant approaches are approved by the FDA, they present distinct advantages and limitations.^189^, ^190^ Effective RNA therapeutics require stable delivery into cells, posing a significant challenge. Delivery methods include non‐viral lipid‐based or polymeric NPs and viral vectors such as adenovirus. While viral vectors offer high transfection efficiency, they may induce immunogenicity and toxicity. In contrast, non‐viral vectors are preferred due to their ease of production, biocompatibility, biodegradability, and reduced immunogenicity.^191^, ^192^

In the context of brain disorders, the BBB presents a major obstacle to RNA therapeutics. The BBB regulates the movement of molecules between the blood and brain, preventing large therapeutic RNAs from crossing. Overcoming this barrier is crucial for developing effective RNA‐based therapies for NDs.^193^, ^194^ Another approach under investigation to address the challenge of BBB penetration is the intracerebral administration of drugs. This method involves directly injecting the drug into the brain through a burr hole drilled in the skull. However, besides its invasive nature, drug distribution is primarily limited to the injection site due to restricted diffusion within the brain. Another solution currently receiving significant attention is nasal delivery to the brain. Small, fat‐soluble molecules can enter the cerebrospinal fluid through the olfactory pathway following nasal administration. Initially, they traverse the nasal epithelial barrier, then the olfactory arachnoid membrane, and finally reach the brain. The primary challenge in this method is the large volumes of therapeutic agents required, which can cause local damage to the nasal membranes.^195^, ^196^

The most promising small molecule regulators targeting ncRNAs in PD are those capable of specifically modulating the expression of miRNAs, lncRNAs, and circRNAs implicated in PD pathogenesis. Among the noteworthy small molecule regulators are miR‐7, miR‐153, and miR‐223. These miRNAs play pivotal roles in PD pathogenesis by binding to the 3′ untranslated region of α‐syn, thereby regulating its expression. They are also proposed as potential PD biomarkers, given their significantly reduced levels in the plasma of PD patients compared to healthy individuals.^197^ miRNAs involved in autophagy regulation, such as miR‐155, miR‐181c, and miR‐214, contribute to PD pathogenesis by influencing α‐syn accumulation, mitochondrial damage, neuroinflammation, and neuronal apoptosis. Targeting these miRNAs holds promise for novel PD therapeutic strategies.^198^ circRNAs are widely expressed in eukaryotes and have essential roles in NDs, including PD. Identification of circRNAs with high specificity and sensitivity may offer new avenues for early PD diagnosis and treatment.^199^ Dysregulation of piRNAs in various PD subtypes and stages suggests their potential as novel biomarkers for PD diagnosis and therapeutic targets.^200^ These small molecule regulators offer promise for modulating the expression of ncRNAs involved in PD pathogenesis, serving as diagnostic biomarkers and therapeutic targets. However, further research is necessary to fully elucidate their roles and develop effective PD treatments.

## 5 CHALLENGES AND FUTURE DIRECTIONS

The advancement of therapies based on ncRNAs in PD entails both obstacles and prospects. Research has shown that some lncRNAs, including HOTAIR and NEAT1, are engaged in the advancement of PD, indicating a possible target for therapy. Nevertheless, the precise function of ncRNAs in PD and the advancement of efficient ncRNA‐based treatments are subjects that are now being actively researched. The research discovered that HOTAIR enhances the incidence of PD by increasing the LRRK2, a gene linked to PD. These findings indicate that focusing on HOTAIR or other lncRNAs might be a viable therapeutic approach.^201^ Furthermore, the use of short ncRNA therapies has demonstrated promise in treating cardiovascular disorders, and analogous strategies could have the capacity to be effective in addressing PD.^202^

Effectively delivering ncRNA therapeutics to the brain poses a considerable challenge. While innovative NPs have been engineered for targeted miRNA delivery in tumors, a similar approach is necessary for ncRNA‐based therapies in PD.^203^ Moreover, comprehending the intricate regulatory networks of ncRNAs in PD is essential for devising efficacious treatments. Recent investigations have delved into the role of competing endogenous RNA (ceRNA) mechanisms in disease progression, offering potential avenues for target identification and early‐stage PD treatment.^81^ The exploration of ncRNAs in neural regeneration represents an intriguing area of study with significant clinical implications. ncRNAs play pivotal roles in the underlying mechanisms of PD and are known to exert essential functional roles in neural regeneration. Although the activation of neural stem/progenitor cells (NSCs) is tightly regulated, it is often insufficient to counterbalance the loss of DopN in PD. Hence, investigating the involvement of ncRNAs in NSC activation and adult neurogenesis following PD onset holds great promise for advancing therapeutic interventions.^204^

Although the promise of ncRNA‐based treatments is gaining more recognition, there are existing limitations that must be tackled. These involve the demand for a more comprehensive comprehension of the distinct functions of various ncRNAs in PD, along with the advancement of efficient delivery techniques for these therapeutic agents.^205^ Moreover, the continuing study aims to identify RNA‐based indicator approaches for PD that have been clinically verified.^206^ Despite the promising potential of therapeutic strategies targeting AAncRNAs, multiple obstacles need resolution. This involves delivery methods for nucleic acid‐based therapeutics, off‐target effects, and the need for precise targeting of specific ncRNAs implicated in PD pathogenesis. Future research efforts should focus on elucidating the complex regulatory networks involving AAncRNAs in PD and developing safe and effective therapeutic interventions.

Numerous innovative initiatives have been devoted to exploring the potential of ncRNAs in clinical trials, particularly in the promising domain of cancer treatment.^207^ However, the landscape of clinical trials focused on ncRNAs and PD remains notably limited, indicating a significant gap that requires extensive further investigation. Nevertheless, the translation of ncRNA research into clinical trials for PD lags. Despite compelling evidence from preclinical studies implicating ncRNAs in PD pathogenesis, progress toward clinical trials has been cautious. Challenges such as elucidating precise molecular mechanisms, establishing reliable biomarkers, and developing effective delivery systems for ncRNA‐based therapies present significant obstacles to clinical translation. Addressing these challenges requires collaborative interdisciplinary efforts encompassing fundamental research, translational investigations, and clinical trials. A thorough understanding of the complex roles of ncRNAs in PD pathology is essential for devising targeted therapeutic approaches. Additionally, innovative strategies to overcome delivery and efficacy barriers associated with ncRNA therapeutics are crucial for unlocking their therapeutic potential in clinical settings.

## 6 CONCLUSION

NDs such as PD are distinguished by defective cellular structures and clumps of proteins in nerve cells, highlighting the crucial function of baseline autophagy in preserving neuronal equilibrium. Studies conducted on mice models with specific ARG deficiencies have shown similarities to ND, highlighting the significance of autophagy in avoiding the accumulation of proteins seen in PD. Furthermore, the clustering of α‐syn hinders the process of autophagy, worsening the harmful effects on the CNS and impairing cellular function. Impaired autophagy worsens the clustering of α‐Syn, hence triggering the development of PD. Gaining an extensive knowledge of these pathways is of utmost importance to design precise therapeutic interventions aimed at reducing NDG in PD.

NcRNAs have complex functions in the development of PD, affecting many signaling systems, including autophagy. Certain miRNAs can enhance autophagy, hence displaying neuroprotective benefits. Conversely, other miRNAs hinder autophagy, exacerbating PD and resulting in neuronal CD. Moreover, lncRNAs have been recognized as crucial determinants in the progression of PD, exerting their influence on crucial mechanisms such as the buildup of α‐Syn protein and the control of autophagy. Acquiring an in‐depth knowledge of the complex interconnection between ncRNAs, autophagy, and the progression of PD is essential for identifying new treatment strategies. CircRNAs are also linked to the course of PD, as research suggests their involvement in the pathogenic pathways of the disease by regulating miRNAs and influencing autophagy. Additional investigation into the circRNA‐mediated modulation of autophagy in PD shows potential for innovative therapeutic approaches.

Therapeutic approaches that focus on ncRNAs involved with autophagy show great potential for the creation of innovative therapeutics for PD. By manipulating the levels and activity of ncRNAs that participate in the control of autophagy, these methods can improve the condition of nerve cells and decelerate the advancement of illness in individuals with PD. Additional preclinical and clinical investigations are necessary to confirm the effectiveness and safety of these treatment strategies and facilitate their use in clinical settings.

## AUTHOR CONTRIBUTIONS

Md Sadique Hussain, Ehssan Moglad, and Muhammad Afzal were involved in concept and design. Gaurav Gupta, Shilpa Sharma, Waleed Hassan Almalki, and Imran Kazmi were involved in literature search and manuscript editing and review. Sami I. Alzarea, G. V. Sivaprasad, Moyad Shahwan, Mahamedha Deorari, and Kumud Pant were involved in conceptual design and writing guidance. Haider Ali, Sachin Kumar Singh, and Kamal Dua were involved in manuscript review. Vetriselvan Subramaniyan edited the manuscript. All authors approved the final version of the review.

## CONFLICT OF INTEREST STATEMENT

The authors declare that there are no competing interests.

## Data Availability

Data sharing is not applicable to this article as no new data were created or analyzed in this study.
